# Is muscle strength an overlooked parameter in patients affected by mild autonomous cortisol secretion?

**DOI:** 10.3389/fendo.2026.1803910

**Published:** 2026-04-30

**Authors:** Martina Romanisio, Chiara Mele, Sara Sturnia, Carola Ciamparini, Rosa Pitino, Alice Ferrero, Lorenza Scotti, Madalina Elena Iftimie, Gianluca Aimaretti, Paolo Marzullo, Flavia Prodam, Marina Caputo

**Affiliations:** 1 Endocrinology, Department of Translational Medicine, Università del Piemonte Orientale, Novara, Italy; 2 Statistics, Department of Translational Medicine, Università del Piemonte Orientale, Novara, Italy; 3 Department of Endocrinology I,’’C.I.Parhon” National Institute of Endocrinology, Bucharest, Romania; 4 Department of Endocrinology, “Carol Davila” University of Medicine and Pharmacy, Bucharest, Romania; 5 Department of Health Sciences, Università del Piemonte Orientale, Novara, Italy

**Keywords:** adrenal, bone, incidentaloma, mild autonomous cortisol secretion, muscle strength

## Abstract

**Introduction:**

Adrenal incidentalomas are an increasing clinical concern and, while most are non-functioning adrenal adenomas (NFAA), a relevant subset is associated with mild autonomous cortisol secretion (MACS), condition linked to various comorbidities, including metabolic and musculoskeletal alterations. However, the association with a reduction in muscle strength and mass is still controversial.

**Methods:**

We evaluated the effects of mild cortisol excess on skeletal muscle health, body composition, and quality of life (QoL) in patients with adrenal incidentalomas associated with MACS, compared with those with NFAA and healthy controls. Sixty-two participants were enrolled: 21 with MACS, 21 with NFAA, and 20 healthy controls. Skeletal muscle strength was assessed using handgrip dynamometry, the sit-to-stand test, and the Medical Research Council (MRC) scale. Body composition was analyzed by bioelectrical impedance analysis (BIA), and quality of life was assessed using the EQ-5D and SARC-F questionnaires.

**Results:**

Patients with MACS had a significantly higher prevalence of osteopenia and osteoporosis compared to NFAA and controls (61.9% vs. 28.6% and 25%, respectively; p= 0.03). No significant differences were observed in fat-free mass (FFM), muscle mass (MM), or fat mass (FM) among groups. MACS patients showed significantly reduced MRC scores for biceps and quadriceps compared to controls (p=0.04). Patients with osteoporosis/osteopenia and reduced muscle strength exhibited higher post-dexamethasone suppression test (DST) cortisol concentrations although not significant. Similarly, QoL scores showed a trend toward greater impairment in the MACS group.

**Conclusion:**

It’s one of the first studies assessing muscle mass and performance in MACS compared to NFAA and healthy controls. Our results underline the impact of mild hypercortisolism on proximal myopathy, suggesting an under-recognized musculoskeletal impact supporting the clinical relevance of MACS.

## Introduction

The widespread use of high-resolution imaging techniques has led to a growing number of diagnoses of adrenal incidentalomas, defined as adrenal masses discovered incidentally during imaging performed for unrelated clinical reasons.

According to the 2016 ESE-ENSAT guidelines (1), non-aldosterone-producing adrenocortical adenomas (NAPACAs), without overt Cushing syndrome (CS), were classified into three categories based on serum cortisol levels after the 1-mg overnight dexamethasone suppression test (DST): autonomous cortisol secretion (ACS) if post-DST cortisol levels were >5 µg/dL, possible autonomous cortisol secretion (PACS), if post-DST cortisol levels were between 1.8-5 µg/dL, and non-functioning adrenal adenoma (NFAA), if post-DST cortisol levels were ≤1.8 µg/dL. In the 2023 ESE-ENSAT guideline update (2), this classification was simplified: patients without overt signs of CS and with post-DST cortisol levels >1.8 µg/dL are now classified as having mild autonomous cortisol secretion (MACS). This change reflects the growing recognition that even mild cortisol excess may have clinical relevance and helps simplify the diagnostic framework used in routine practice (3–9).

Recent original studies and systematic reviews suggest that even mild cortisol excess may be associated with increased cardiometabolic morbidity, cardiovascular risk, and all-cause mortality, while some treatment-related studies indicate possible improvement in selected cardiometabolic outcomes after adrenalectomy in carefully selected patients with MACS (10–15). Although these patients often lack overt signs of CS, hypercortisolism is a well-documented cause of skeletal muscle atrophy and weakness, especially in the proximal lower limbs. Multiple glucocorticoid-driven mechanisms, including reduced protein synthesis, activation of proteolytic pathways, mitochondrial dysfunction, and impairment of neuromuscular transmission, mediate these effects (16–18). While the pathophysiological basis of glucocorticoid-induced myopathy is well established in overt CS, whether similar (albeit subtler) alterations are present in individuals with MACS remains unclear.

Frailty, characterized by a decline in physiological reserves and functional capacity, leading to adverse health outcomes, could be considered a marker of vulnerability in this population. It can be assessed through validated tools including the Frailty Index or performance-based physical assessments (19). In contrast, performance-based measures provide objective assessments of muscle strength and physical function, typically via handgrip strength, gait speed, or the chair rise test (20). Recent evidence from a multicenter prospective study demonstrated that patients with incidentally discovered adrenal adenomas—whether associated with MACS or NFAA—exhibit a significantly higher burden of frailty compared to general population. Moreover, the prevalence of frailty increased progressively with higher DST cortisol levels and Frailty Index showed a strong correlation with performance-based measures (21).

Despite its prognostic relevance, muscle function remains under-investigated in routine clinical practice. However, it is a sensitive marker of early functional decline and a well-established predictor of poor outcomes. In individuals with MACS, the absence of overt signs of hypercortisolism often delays diagnosis, resulting in prolonged exposure to subclinical cortisol excess, which may contribute to accelerated functional deterioration and premature aging phenotype (22).

The present study aims to evaluate muscle strength and physical performance in patients with MACS, compared with individuals with NFAA and healthy controls. By integrating body composition analysis, functional assessments, and patient-reported outcomes, this study seeks to better characterize the functional consequences of MACS and to identify early markers of clinical impairment in these patients.

## Patients and methods

### Study design

This cross-sectional case-control study was conducted from May 2024 to January 2025. Adult patients with newly diagnosed NAPACAs and healthy subjects were recruited from the Endocrinology Unit at “Maggiore della Carità” Hospital in Novara, Italy.

Data collection included medical history, physical examination, body composition analysis, laboratory tests, muscle strength assessment, and psychological evaluation using standardized questionnaires.

The investigator who performed muscle strength assessments [handgrip strength test, Sit-to-Stand test, and Medical Research Council scale (MRC) scale] was blinded to the hormonal status of the participants at the time of evaluation, in order to minimize potential assessment bias.

The study was approved by the Local Ethics Committee of Maggiore della Carità Hospital (CE 271/2024) and conducted in accordance with the Declaration of Helsinki and its amendments. All patients provided written informed consent.

### Participants

The study population includes three groups matched by age, sex, and BMI: i) patients with adrenal adenomas associated with MACS, ii) patients with NFAA, iii) healthy controls. Inclusion criteria were: (1) age ≥18 years, (2) ability to complete study questionnaires, and (3) diagnosis of adrenal adenoma >1 cm (NAPACAs) for patient groups.

The exclusion criteria were: (1) conditions affecting muscle strength (e.g., active malignancy undergoing chemotherapy, thyroid dysfunction, chronic steroid therapy, previous motor disabilities); (2) diagnosed CS, adrenal carcinoma, primary hyperaldosteronism, or pheochromocytoma; (3) conditions significantly affecting cortisol levels (e.g., severe chronic inflammatory/autoimmune diseases, severe liver or kidney failure, and treatment with glucocorticoid/immunomodulatory therapy).

Control subjects were identified during an endocrine outpatient evaluation in the recruitment period, among healthy individuals evaluated for conditions unrelated to adrenal disease. All selected control subjects had undergone abdominal ultrasonography, non-contrast computed tomography (CT), and/or magnetic resonance imaging (MRI) for other clinical reasons, with no evidence of adrenal lesions on imaging within the previous five years and no clinical conditions known to affect muscle strength. When the most recent CT scan was older than two years, a 1-mg DST was performed to exclude cortisol excess. After the initial screening, definitive control participants were selected according to predefined matching criteria (age ± 2 years, sex, and BMI) to ensure comparability with the MACS and NFAA groups. Individuals who did not meet these matching criteria were not included in the final analysis.

### Hormonal, biochemical and imaging evaluations

Patients with adrenal incidentalomas were assessed according to the 2023 ESE/ENSAT guidelines (2). Benignity was confirmed via non-contrast CT and/or MRI.

All patients underwent 1-mg DST. MACS was defined by a post-DST cortisol level >1.8 µg/dL in the absence of CS stigmata (e.g., facial plethora, supraclavicular fat accumulation, buffalo hump, striae rubrae, easy bruising). In these patients, plasma adrenocorticotropic hormone (ACTH) levels were measured between 8:00-9:00 am to exclude ACTH-dependent hypercortisolism.

In patients with hypertension or unexplained hypokalemia, the aldosterone-to-renin ratio (ARR) was assessed to exclude primary hyperaldosteronism, following guideline cut-offs (23). If the suspicion was high, patients were excluded.

Fasting plasma glucose, glycated hemoglobin (HbA1c), lipid profile, total calcium, and 25-hydroxy vitamin D (25(OH)D) levels were evaluated.

Comorbidities (i.e., impaired glucose metabolism, arterial hypertension, dyslipidemia, osteoporosis/osteopenia, and atrial fibrillation) were recorded and diagnosed according to standard guidelines.

### Muscle function assessment

#### Hand grip strength test

Hand grip strength was assessed using a Jamar Hydraulic Hand Dynamometer. Participants were seated with their feet flat on the floor and elbow flexed at 90° and instructed to squeeze the dynamometer with maximum effort while keeping their trunk and arm stationary. The test was repeated three times, and the highest value used for analysis. Sarcopenia was defined according to the 2019 EWGSOP2 criteria: grip strength <27 kg for men and <16 kg for women (24).

#### Sit-to-stand test

Lower limb strength, particularly the quadriceps, was evaluated using the Sit-to-Stand test. Participants were timed while completing five unassisted rises from a seated position. Scores were assigned on a scale from 0 (unable to complete the test) to 4 (completed in less than 11.2 seconds).

#### MRC scale

The MRC scale was used to assess isometric resistance in the biceps brachii and quadriceps femoris. Strength was graded on a six-point scale: 5) normal strength, 4) movement possible against moderate resistance, 3) movement possible against gravity but not against resistance, 2) movement possible in the absence of gravity, 1) visible contraction with no movement, 0) no contraction.

#### Body composition assessment

Body composition was measured using bioelectrical impedance analysis (BIA) with the TANITA MC-780MA device. This multifrequency segmental analyzer automatically provided estimates for: weight and BMI, total free fat mass (FFM, kg), total muscle mass (MM, kg and %), total fat mass (FM, kg and %), and skeletal muscle mass of the arms and legs. Raw impedance and reactance data are not available in this software.

Appendicular skeletal muscle mass (ASM) was calculated as the sum of muscle mass in the upper and lower limbs. The Appendicular skeletal muscle index (ASMI) was determined by adjusting ASM for height squared (ASM/height2), expressed in kg/m2. Visceral fat levels were categorized as moderate: < 12, high: between 13 and 18 and very high: > 18. Additional parameters automatically extracted included: body water distribution (total, intracellular, and extracellular water - kg and %), sarcopenic index (kg/m2), phase angle (°), and basal metabolic rate (BMR) (kcal/day).

### Questionnaires

Participants completed three self-reported questionnaires: SARC-F, EQ-5D and Numerical Rating Scale (NRS). The SARC-F questionnaire was used as a screening tool to assess sarcopenia risk. The EQ-5D served as a general measure of health-related quality of life. Pain intensity, including chronic pain conditions, was evaluated using the Numerical Rating Scale (NRS), an 11-point scale ranging from 0 (no pain) to 10 (worst imaginable pain). The type and amount of physical activity of each participant were investigated by IPAQ questionnaire (25–28).

### Statistical analysis

Continuous variables are expressed as mean ± standard deviation (SD) if normally distributed, or as median with interquartile ranges (IQR) if non-normally distributed. Categorical variables are reported as absolute numbers and percentages. Normality was assessed using the Shapiro–Wilk test; non-normally distributed variables were log-transformed to improve symmetry and homoscedasticity.

Descriptive statistics were applied to the total sample and stratified by group.

Comparisons between subgroups (MACS, NFAA, and healthy controls) were performed using one-way ANOVA for normally distributed continuous variables, the Kruskal–Wallis test for non-normally distributed continuous variables, and the chi-square test for categorical variables. When comparisons involved only two groups, the Mann–Whitney U test was applied.

After excluding healthy controls, correlations between cortisol levels and clinical, demographic, and lifestyle variables were assessed using Spearman’s rank correlation coefficient.

Based on a one-way ANOVA design with three groups and equal group allocation, a total sample size of 60 subjects (20 per group) was calculated to provide 80% power at a two-sided α level of 0.05 to detect a clinically meaningful difference in handgrip strength of approximately 5 kg among groups, corresponding to a moderate-to-large effect size (Cohen’s f≈0.40) (29).

A p-value < 0.05 was considered statistically significant. All analyses were conducted using SPSS software, version 27 (IBM Corp., Armonk, NY, USA).

## Results

### Demographic and clinical characteristics

**Figure 1:**
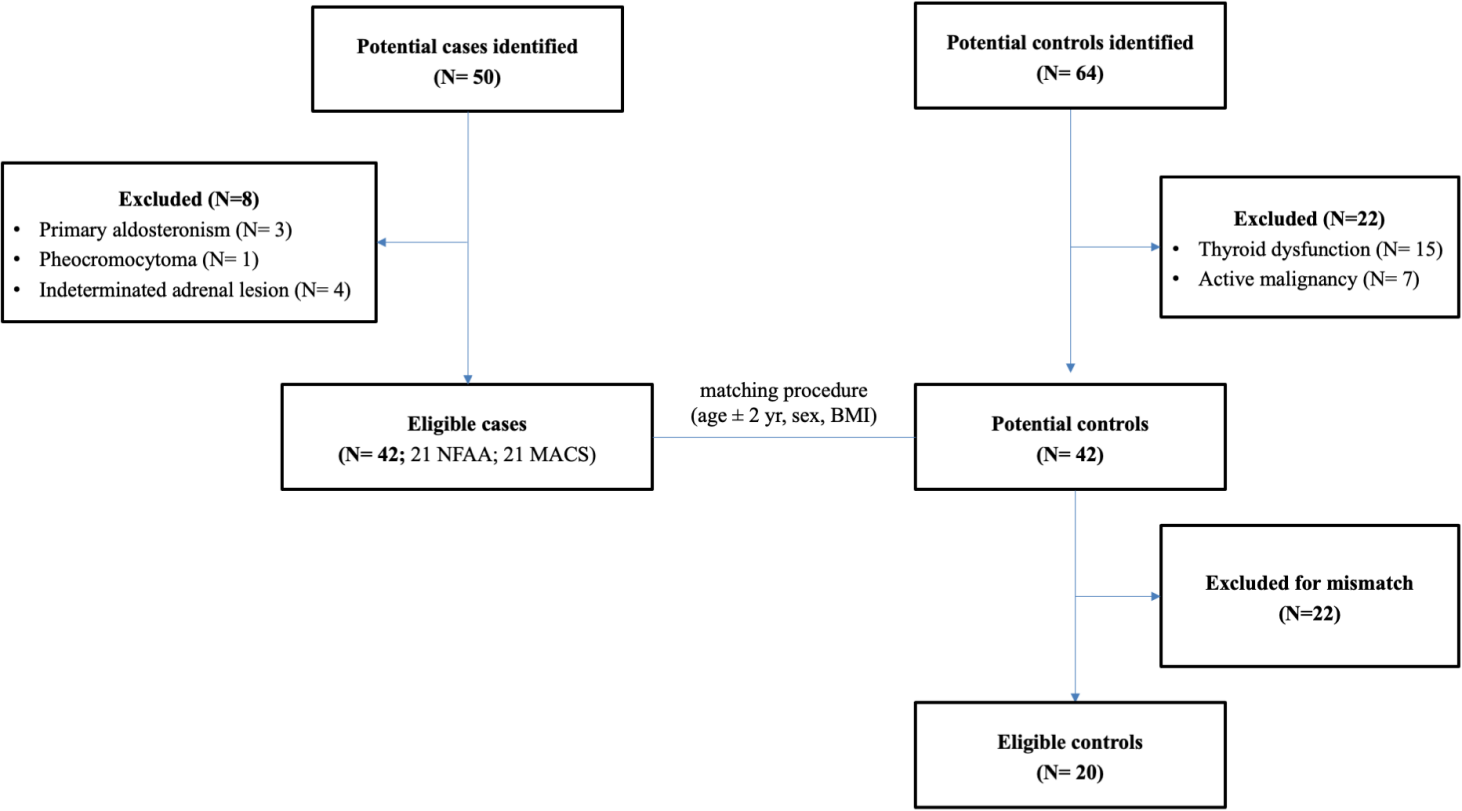
Enrolment process.

Fifty patients with NAPACAs were initially screened. Following hormonal and imaging evaluation, 8 patients were excluded (3 with primary aldosteronism, 1 with suspected pheochromocytoma, and 4 with indeterminate adrenal lesions). Thus, 42 cases were included in the final analysis (21 MACS, 21 NFAA). Sixty-four potential controls were identified, and, after application of exclusion criteria (15 for thyroid dysfunctions and 7 for active malignancy), 42 potential controls were recruited. Following matching for age (± 2 years), sex and BMI, 20 matched controls were included in the analysis (**Figure 1**).

The mean age was 69.1 ± 7.2 years in the MACS group, 68.1 ± 8.9 years in the NFAA group, and 69.4 ± 9.1 years in the control group. Females represented 57.1% of participants in both the MACS and NFAA groups, and 60.0% in the control group. A detailed summary of the demographic and clinical parameters is provided in **Table 1**.

**Table 1:** Demographic, clinical and hormonal characteristics of enrolled patients, divided by groups.

Variables	Total	MACS	NFAA	Control group	ANOVA P value
	(n = 62)	(n = 21)	(n = 21)	(n = 20)	
Age, years, mean ± SD	68.8 ± 8.3	69.1 ± 7.1	68 ± 8.9	69.4 ± 9.1	0.8610
Sex, F, n (%)	36 (58.1)	12 (57.1)	12 (57.1)	12 (60)	0.9775^
Smoker					
Current, n (%)	5 (8.1)	2 (9.5)	1 (4.8)	2 (10.0)	0.4877*
Past, n (%)	2 (3.2)	2 (9.5)	0 (0.0)	0 (0.0)	
Comorbidities					
T2D, n (%)	9 (14.5)	3 (14.3)	4 (19)	2 (10)	0.2785*
IFG, n (%)	15 (24.2)	6 (28.6)	7 (33.3)	2 (10)	
AH, n (%)	43 (69.4)	16 (76.2)	16 (76.2)	11 (55)	0.2390^
Dyslipidemia, n (%)	41 (66.1)	15 (71.4)	15 (71.4)	11 (55)	0.4421^
Osteoporosis, n (%)	6 (9.7)	4 (19.0)	0 (0)	2 (10)	0.0362*
Osteopenia, n (%)	18 (29)	9 (42.9)	6 (28.6)	3 (15)	
AF, n (%)	1 (1.6)	0 (0)	0 (0)	1 (5)	0.3226*
Adrenal adenoma					
Size, median (Q1-Q3)	21 (17-26)	22 (20-28)	20 (15-23)		0.0850^#^
Bilateral, yes, n (%)	10 (23.81)	5 (23.8)	5 (23.8)		1.0000^
Cortisol after 1-mg DST, mcg/dl, median (Q1-Q3)	1.6 (0.9-2.8)	2.8 (2.1-3.5)	1.4 (1.1-1.6)	0.7 (0.5-0.9)	**<0.0001**
ACTH, pg/ml, mean ± SD		8.77 ± 4.24			

^chi square test, *Fisher’s exact test, °Kruskall-Wallis test, ^#^Mann-Whitney test ACTH, Adrenocorticotropic hormone, AF, atrial fibrillation, AH, arterial hypertension; DST, dexamethasone, IFG, impaired fasting glucose, MACS, mild autonomous cortisol secretion, NFAA, non-functioning adrenal adenomas, T2D, type 2 diabetes mellitus. The significant values are shown in bold..

Among NAPACAs, the median adenoma diameter was 21 mm (IQR 17–26), with bilateral lesions observed in 10 cases (23.8%), equally distributed between MACS and NFAA subgroups. As expected, post-DST cortisol levels were higher in MACS compared to NFAA, indicating modest cortisol hypersecretion, according to the inclusion criteria (p<0.0001).

Considering comorbidities, no significant differences were found between groups regarding the prevalence of type 2 diabetes (T2D), impaired fasting glucose (IFG), arterial hypertension, dyslipidemia, or atrial fibrillation (AF). Osteoporosis and osteopenia were more frequently observed in the MACS (61.9%) compared to NFAA (28.6%) and controls (25.0%) (p=0.03).

### Anthropometric and metabolic parameters

Anthropometric and metabolic parameters are summarized in **Table 2**. BIA analysis revealed no significant differences among groups in FM, FFM, MM, appendicular skeletal muscle mass (ASM), or phase angle, even after adjusting for BMI.

**Table 2:** Anthropometric and metabolic parameters of patients, divided by groups.

Variables	Total	MACS	NFAA	Control group	ANOVA P value
	(n = 62)	(n = 21)	(n = 21)	(n = 20)	
Fasting plasma glucose, mg/dl, median (Q1-Q3)	95 (90-101)	95 (87-104)	95 (90-103)	93 (90-97)	0.6312°
HbA1c, %, mean ± SD	5.77 ± 0.38	5.82 ± 0.39	5.6 ± 0.34	5.9 ± 0.42	0.3330
Total cholesterol mg/dl, mean ± SD	172.35 ± 41.84	178.05 ± 44.31	166.06 ± 40.95	172.75 ± 41.41	0.6923
c-HDL, mg/dl, media ± SD	56.24 ± 14.32	61.11 ± 18.23	51.28 ± 11.34	56 ± 8.44	0.1121
Triglycerides, mg/dl, median (Q1-Q3)	94 (81-131)	97 (80-129.5)	90 (81.25-128.5)	98 (81.75-133)	0.9986°
c-LDL, mg/dl, mean ± SD	94.87 ± 34.05	93.44 ± 34.10	94.68 ± 34.08	97.67 ± 36.74	0.9445
Calcium, mg/dl, mean ± SD	9.59 ± 0.44	9.64 ± 0.56	9.62 ± 0.33	9.51 ± 0.4	0.6767
25OH Vitamin D, ng/ml, median (Q1-Q3)	27.9 (21.1-34.8)	23.6 (19.15-31.35)	31 (22.9-51.35)	27.6 (25.4-31.1)	0.1332°
BMI, kg/m^2^, mean ± SD	29.11 ± 5.57	29.11 ± 6.27	30.11 ± 5.37	28.06 ± 5.07	0.5042
FFM, kg, median (Q1-Q3)	51.3 (46.2-64.6)	49.7 (47.2-64)	50.9 (45.5-65.3)	52.05 (47.3-59.85)	0.9238°
FFM, %, mean ± SD	69.97 ± 8.49	69.94 ± 9.07	68.81 ± 6.96	71.23 ± 9.51	0.6673
MM, %, mean ± SD	66.43 ± 8.08	66.39 ± 8.64	65.34 ± 6.64	67.61 ± 9.05	0.6763
ASM, kg, median (Q1-Q3)	20.2 (18.3-25.7)	20.7 (18.4-24.5)	19.9 (17.9-28)	20.15 (18.73-24)	0.9467°
ASMI, kg/m^2^, mean ± SD	8.07 ± 1.32	8.08 ± 1.56	8.3 ± 1.42	7.8 ± 0.88	0.4712
UL-ASM, kg, median (Q1-Q3)	5.2 (4.6-6.8)	5.3 (4.8-6.7)	5.2 (4.6-7.8)	5.2 (4.7-6.3)	0.9112°
LL-ASM, kg, median (Q1-Q3)	15.1 (13.6-18.9)	15.2 (13.6-18.1)	14.7 (13.3-20.1)	15 (14.05-17.7)	0.9477°
FM, %, mean ± SD	30.04 ± 8.49	30.08 ± 9.08	31.19 ± 6.96	28.78 ± 9.52	0.6699
A-FM, %, mean ± SD	48.97 ± 9.37	47.8 ± 9.38	50.73 ± 9.6	48.35 ± 9.34	0.5685
V-FM, level	11 (9-15)	11 (9-15)	11 (9-16)	11 (8.75-13.5)	0.7713°
FM(%)/FFM(%), mean ± SD	0.45 ± 0.18	0.45 ± 0.19	0.47 ± 0.16	0.43 ± 0.2	0.7948
Sarcopenic INDEX (kg/m2), mean ± SD	8.05 ± 1.31	8.06 ± 1.54	8.28 ± 1.41	7.79 ± 0.88	0.4934
ECW/TBW,%, mean ± SD	45.17 ± 2.43	45.24 ± 2.26	45.02 ± 2.52	45.27 ± 2.63	0.9400
Phase angle, °, mean ± SD	5.64 ± 1.04	5.4 (5-5.8)	5.6 (5.1-6)	5.4 (4.9-5.83)	0.5836°
Basal metabolism, kcal, median (Q1-Q3)	1527.5 (1385-1854)	1496 (1404-1848)	1527 (1343-2014)	1528.5 (1403.75-1735)	0.9016°

^chi square test, *Fisher’s exact test, °Kruskall-Wallis test, ^#^Mann-Whitney test BMI, body mass index, FFM, fat free mass, MM, muscle mass, ASM, appendicular skeletal mass, ASMI, appendicular skeletal mass index, UL-ASM, upper limb ASM, LL-ASM, lower limb ASM, FM, fat mass, A-FM, abdominal FM, V-FM, visceral FM, ECW, extra cellular water, TBW, total body water. The significant values are shown in bold.

Metabolic and osteometabolic profiles were also similar across groups. Twenty-nine percent of participants were receiving cholecalciferol supplementation. Among the 42 subjects with available 25(OH)D data, 16 (38.1%) had sufficient vitamin D levels (>30 ng/mL), whereas 26 (61.9%) had non-sufficient levels. Among those with non-sufficient vitamin D levels, 16/26 (61.5%) had mild insufficiency (20-29.9 ng/mL). Among participants with osteopenia or osteoporosis, 20 had available 25(OH)D data; in this subgroup, the median 25(OH)D concentration was 30.5 ng/mL (IQR 22.3-45.3), 10/20 (50.0%) had sufficient vitamin D levels, and, among those with non-sufficient levels, 6/10 (60.0%) had mild insufficiency.

### Muscle strength and functional assessments

In all patients the right limb was the dominant one. Manual muscle testing using the MRC scale revealed a higher prevalence of proximal muscle weakness in patients with MACS (**Table 3**). Specifically, reduced strength of the right biceps brachii (dominant upper limb) was observed in 23.8% of patients with MACS, compared to 4.8% in the NFAA group and none in controls (p=0.04). Similarly, reduced quadriceps femoris strength was detected in 38.1% of MACS, 28.6% of NFAA, and 5.0% of controls (p=0.03).

**Table 3:** Muscle strength and functional assessment evaluated by MRC scale, Sit-to-Stand Test and dynamometry of enrolled patients, divided into groups.

Variables	Total	MACS	NFAA	Control group	ANOVA P value
	(n = 62)	(n = 21)	(n = 21)	(n = 20)	
MRC SCALE – right biceps brachii					
Movement possible against moderate resistance, n (%)	6 (9.68)	5 (23.81)	1 (4.76)	0 (0.00)	0.0401*
Normal force, n (%)	56 (90.32)	16 (76.19)	20 (95.24)	20 (100.00)	
MRC SCALE – left biceps brachii					
Movement possible against moderate resistance, n (%)	7 (11.29)	4 (19.05)	2 (9.52)	1 (5.00)	0.4805*
Normal force, n (%)	55 (88.71)	17 (80.95)	19 (90.48)	19 (95.00)	
MRC SCALE – right quadriceps femori					
Movement possible against gravity but not against resistance, n (%)	1 (1.61)	0 (0.00)	1 (4.76)	0 (0.00)	0.0397*
Movement possible against moderate resistance, n (%)	14 (22.58)	8 (38.10)	5 (23.81)	1 (5.00)	
Normal force, n (%)	47 (75.81)	13 (61.90)	15 (71.43)	19 (95.00)	
MRC SCALE – left quadriceps femori					
Movement possible against gravity but not against resistance, n (%)	1 (1.61)	0 (0.00)	1 (4.76)	0 (0.00)	0.5898*
Movement possible against moderate resistance, n (%)	19 (30.65)	6 (28.57)	8 (38.10)	5 (25.00)	
Normal force, n (%)	42 (67.74)	15 (71.43)	12 (57.14)	15 (75.00)	
DYNAMOMETRY – right upper arm					
Normal muscle strength, n (%)	46 (74.19)	17 (80.95)	14 (66.67)	15 (75.00)	0.5686^
Reduced muscle strength, n (%)	16 (25.81)	4 (19.05)	7 (33.33)	5 (25.00)	
Value – right upper arm, median (Q1-Q3)	21.5 (18-30)	22 (19-27)	21 (19-28)	22 (18-34.5)	0.9037°
DYNAMOMETRY – left upper arm					
Normal muscle strength, n (%)	42 (67.74)	15 (71.43)	13 (61.90)	14 (70.00)	0.7770^
Reduced muscle strength, n (%)	20 (32.26)	6 (28.57)	8 (38.10)	6 (30.00)	
Value - left upper arm, median (Q1-Q3)	20 (16-30)	22 (16-30)	23 (15-28)	19 (17-34)	0.9747°
SIT TO STAND TEST					
>16,6’’, n (%)	8 (12.90)	2 (9.52)	3 (14.29)	3 (15.00)	0.7901*
16,6’’-13,7’’, n (%)	10 (16.13)	4 (19.05)	4 (19.05)	2 (10.00)	
13,6’’-11,2’’, n (%)	16 (25.81)	7 (33.33)	3 (14.29)	6 (30.00)	
<11,2’’, n (%)	28 (45.16)	8 (38.10)	11 (52.38)	9 (45.00)	
SARC-F					
Low probability of sarcopenia, n (%)	56 (90.32)	17 (80.95)	20 (95.24)	19 (95.00)	0.3458*
High probability of sarcopenia, n (%)	6 (9.68)	4 (19.05)	1 (4.76)	1 (5.00)	
Value, median (Q1-Q3)	1 (0-3)	2 (0-3)	1 (0-2)	1 (0-2)	0.1660
IPAQ, level of physical activity, n (%)					0.7348
Low	2 (3.2)	1 (4.8)	0 (0)	1 (5)	
Moderate	59 (95.2)	20 (95.2)	20 (95.2)	19 (95)	
High	1 (1.6)	0 (0)	1 (4.8)	0 (0)	

^chi square test, *Fisher’s exact test, °Kruskall-Wallis test, ^#^Mann-Whitney test.

Reduced muscle strength was defined according to EWGSOP2 criteria (handgrip strength <27 kg in men and <16 kg in women). MRC scale, Medical Research Council scale, IPAQ, International Physical Activity Questionnaire. The significant values are shown in bold.

In contrast, quantitative assessments of muscle strength and function – performed using handgrip dynamometry and the Sit-to-Stand test - did not show significant differences among groups. The probability of sarcopenia, screened by the SARC-F questionnaire, was low across all groups, although slightly higher in MACS (19.1%) compared to NFAA (4.8%) and controls (5.0%), without reaching significance.

These results were confirmed even after correction for level of physical activity assessed by IPAQ questionnaire, and for 25(OH)D level.

### Quality of life and pain assessment

Health-related quality of life, assessed using the EQ-5D questionnaire, revealed a non-significant trend toward functional impairment in the MACS group (**Supplementary Table 1**). Mobility limitations were reported by 38.1% of MACS patients, compared to 25.8% in the NFAA group and 20% in controls. Difficulties in self-care were noted in 14.3% of MACS patients, 4.8% of NFAA, and none of the controls, while limitations in performing usual activities were reported by 38.1% of MACS, 28.6% of NFAA, and 10% of controls. Contrary, the NFAA group showed a higher frequency of anxiety (61.9%), followed by MACS (42.85%) and controls (25.0%), although this difference did not reach statistical significance. While pain frequency was similar across groups, patients with adrenal adenomas reported slightly higher median pain intensity, as measured by the Numerical Rating Scale (NRS). Self-rated overall health status, assessed via the EQ-5D, was lower in the NAPACA patients (MACS or NFAA) (median 70; IQR 65–80) compared to controls (median 80; IQR 70–85), though not significant.

### Cortisol and clinical correlates

Post-DST cortisol levels were analyzed in relation to comorbidities and clinical features (**Supplementary Table 2**).

**Figure 2:**
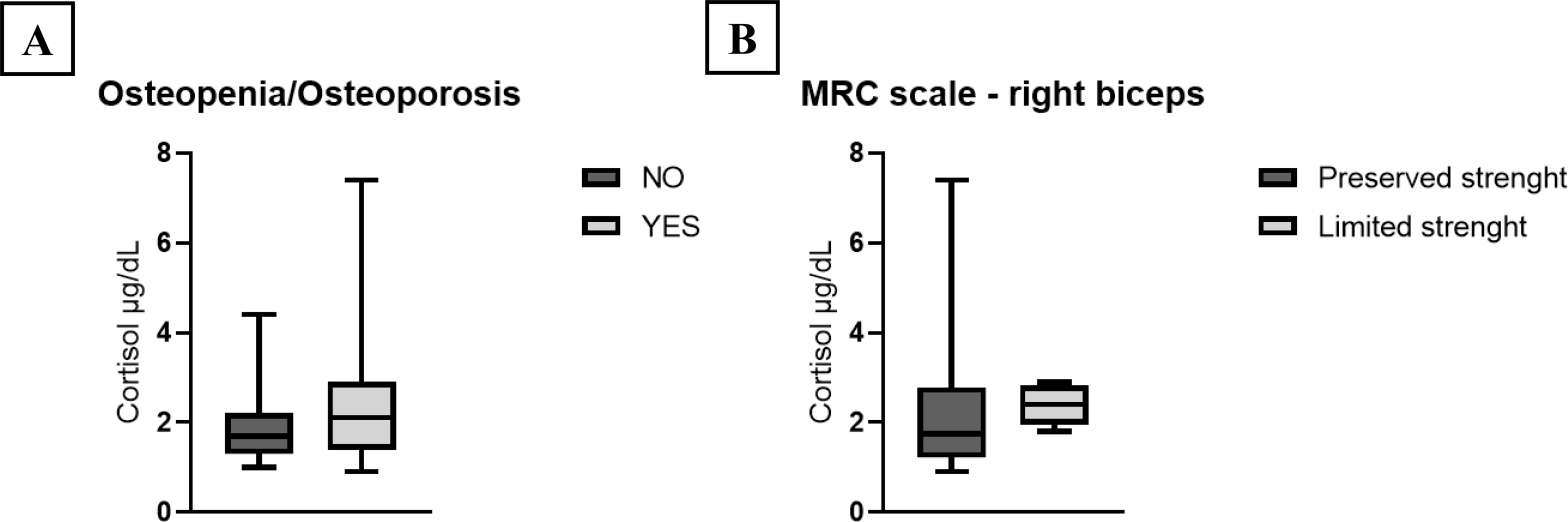
**(A)** Post-DST cortisol levels according to the presence or absence of bone metabolic disease (osteopenia/osteoporosis); **(B)** Post-DST cortisol levels according to muscle strength of the right biceps, assessed by the MRC scale. Data are shown as box-and-whisker plots representing the median, interquartile range, and outliers.

Patients with osteoporosis or osteopenia exhibited a trend toward higher cortisol levels compared to those without bone metabolism disorders, although not significant (**Figure 2A**).

Higher post-DST cortisol concentrations were also observed in individuals with reduced muscle strength, although again not significant (**Figure 2B**). Similarly, participants reporting mobility limitations or pain had slightly elevated cortisol levels (median 2.20 µg/dL; IQR 1.80–2.90) compared to those without such symptoms (median 1.70 µg/dL; IQR 1.40–2.20).

No significant correlations were observed between post 1-mg DST and BMI, FFM, FM, ASM, or ASMI.

25OH-D levels showed a trend toward a negative correlation with post-DST cortisol levels (rho=−0.32, p=0.06).

## Discussion

MACS, formerly referred to as subclinical CS, is associated with subtle but clinically relevant metabolic comorbidities, as well as an increased risk of vertebral fractures (2). Although cortisol levels in MACS are only modestly elevated, the chronicity of exposure can lead to cumulative detrimental effects across multiple organ systems, burdening the clinical management of patients and influencing therapeutic decisions, including the potential indication for adrenalectomy (2). Whether mild cortisol excess is causally associated with reduced muscle strength and mass in MACS is still controversial.

Muscle mass maintenance is negatively regulated by cortisol, which promotes protein breakdown and inhibits protein synthesis via glucocorticoid receptors (GRs). Skeletal muscle-specific GR-knockout mice have increased muscle mass (30). In humans, observational studies reported that mild cortisol excess characteristic of aging (31–33) and chronic stress (34), is associated with a reduced muscle strength and mass (35), detrimental physical performance (36), and, in turn, the presence of sarcopenia (37, 38), although results have not been reproduced by all authors (39). However, a recent Mendelian Randomization study designed to investigate the association between cortisol and grip strength on 12,597 individuals of the CORtisol NETwork (CORNET) consortium of 11 Western European population-based cohorts, demonstrated that the increase in cortisol was associated with reduction in grip strength, whole-body lean mass and appendicular lean mass in women (40).

In line with this evidence, in our study muscle strength assessment revealed that patients with MACS were more likely to exhibit deficits on the MRC scale than NFAA and controls, particularly in the dominant upper and lower limbs. Our results are partially consistent with a study that specifically investigated the role of endogenous hypercortisolism on muscle strength (41), demonstrating muscle weakness and reduced quality of life in overt CS and MACS. In that study, the sit-to-stand test appeared more frequently impaired than handgrip strength. However, in our cohort, neither the sit-to-stand test nor handgrip dynamometry demonstrated significant differences between groups. This discrepancy may be related to the relatively mild degree of cortisol excess in our population, the limited sample size, or methodological differences between studies. Notably, in our study, muscle weakness was primarily detected through the MRC scale rather than performance-based tests. Therefore, our findings should be interpreted with caution. Rather than indicating a generalized reduction in muscle strength, they suggest that mild cortisol excess may be associated with subtle proximal muscle weakness that is more readily captured by focused clinical examination, reflecting the predominant impact of hypercortisolism on proximal muscles (biceps brachii and quadriceps femoris) (42).

While several studies investigated muscle strength in overt CS (43, 44), few studies evaluated muscle strength in patients with MACS (21, 29). The first study that compared muscle mass in MACS and NFAA demonstrated that 1-mg DST cortisol levels were inversely correlated with appendicular skeletal muscle mass (ASM), lower limb ASM, and appendicular skeletal muscle index in women, but not men, suggesting that MACS patients had lower skeletal muscle mass with a sex-related effect, although the muscle functionality was not assessed (45). Another recent cross-sectional study demonstrated that hand grip strength decreased in patients with adrenal incidentalomas compared to healthy controls, and this trend was more pronounced in MACS group (29).

To date, this is one of the first studies that assessed muscle mass and performance in MACS compared to NFAA and healthy controls. Although no statistically significant, patients with reduced strength tended to exhibit higher post-DST cortisol concentrations, reinforcing the hypothesis of a cortisol-mediated impact on muscle functionality.

Bioimpedance analysis did not detect significant differences between groups in FM, FFM or ASM; nonetheless, MACS patients exhibited a trend toward increased visceral adiposity and reduced muscle mass. These observations, though limited by bioimpedance’s sensitivity relative to more advanced imaging modalities as abdominal CT or dual-energy X-ray absorptiometry (DXA), align with a recent extensive study by Kim et al., which examined over 2,900 patients with adrenal tumors. The Authors showed as individuals with MACS and NFAA exhibited similar, yet still unfavorable, body compositions compared to healthy controls. In particular, NFAA and MACS patients had greater visceral fat area and lower muscle and adipose attenuation on CT scans than controls (45). Importantly, mild glucocorticoid excess in MACS may induce a state of relative hyperhydration and alterations in body water distribution. Since bioelectrical impedance analysis estimates lean mass based on tissue conductivity and hydration status, such changes may lead to an overestimation of muscle mass, potentially masking underlying sarcopenia. This methodological limitation may partly explain the discrepancy between our findings and those reported by Kim et al., who identified reduced muscle mass in MACS using imaging-based techniques rather than impedance-derived measurements (14). Interestingly, visceral fat accumulation itself has been proposed as a potential contributor to the development of adrenal incidentalomas, suggesting a possible bidirectional relationship between adiposity and adrenal tumor biology (46).

Notably, proximal muscle weakness in patients with MACS was detectable despite preserved muscle mass on BIA. This finding suggests that functional impairment may precede measurable quantitative muscle loss in the context of mild cortisol excess (47). In this setting, cortisol-related alterations in muscle quality, neuromuscular transmission, or mitochondrial function may occur before significant reductions in total muscle mass are measurable. These findings may suggest that focused clinical assessment of proximal muscle strength could help detect subtle functional impairment in MACS. However, because significant differences were not consistently observed across all functional tests, this interpretation should be considered preliminary.

Bone fragility emerged as one of the most prominent clinical features in our MACS cohort. Assessment of bone mineral density by DXA is a suboptimal tool to diagnose glucocorticoid-induced osteoporosis since cortisol excess has a greater impact on bone microarchitectural quality than on bone mineral density (48, 49). Furthermore, conflicting results on bone mineral density in patients with MACS have been reported, with significant findings, particularly when bone microarchitecture was assessed qualitatively using methods such as the trabecular bone score (TBS) (50). A recent meta-analysis and regression of 16 observational studies confirmed not only a higher likelihood of any fractures (vertebral and non-vertebral) in patients with MACS than NFAA, but also of osteoporosis/osteopenia. In particular, subjects with MACS had significantly lower bone mineral density at lumbar spine and femoral neck than NFAA (51).

In our cohort, osteopenia and osteoporosis were significantly more prevalent in MACS patients than in NFAA and controls, despite the relatively mild degree of hypercortisolism (median post-DST cortisol 2.8 µg/dL). While the correlation between cortisol levels and bone disease did not reach statistical significance, a clear trend was observed: higher cortisol levels in patients with osteopenia or osteoporosis. A recent multicenter cross-sectional study involving patients with NFAA, MACS, CS and subjects without adrenal disorders demonstrated that both patients with CS and MACS had a higher prevalence of osteoporosis when compared to referents and, among patients with MACS, only those with post 1-mg DST cortisol > 3 mcg/dl had a higher prevalence of osteoporosis when compared to referents (52). Another Mendelian randomization study demonstrated a significant causal relationship between cortisol levels and the progression of osteoporosis in specific regions, as femoral neck; moreover, the 2 step Mendelian randomization approach showed that sarcopenia mediates up to 8.4% of the cortisol-induced osteoporosis risk in the lumbar spine, highlighting the substantial mediating role of sarcopenia between cortisol and lumbar osteoporosis (53).

The main limitations of the study are the narrow range of cortisol values and the relatively small sample size, which could have limited the statistical power to detect meaningful associations. In particular, because all patients with MACS were incidentally diagnosed during exams for extra-adrenal causes, the duration of mild cortisol excess not assessable. Second, the accuracy of BIA in estimating muscle mass remains debated, despite its widespread use as a non-invasive and cost-effective tool for body composition assessment. Importantly, BIA does not provide information on muscle quality, including fatty infiltration (myosteatosis) or intramuscular lipid accumulation. This methodological limitation may partly account for the discrepancy observed between preserved muscle mass and impaired muscle strength in patients with MACS. In this context, more advanced imaging techniques, such as CT, MRI, or DXA-derived muscle quality parameters, may allow a more comprehensive characterization of cortisol-related musculoskeletal alterations.

Moreover, given the impact of cortisol on bone microarchitectural texture, integrating advanced tools such as TBS may enhance the identification of patients at increased risk (48). Finally, given the sample size and the limited number of participants within each subgroup, we were unable to perform sex-stratified analyses with adequate statistical power. Considering prior evidence of sex-related differences in cortisol-associated muscle and bone outcomes (54, 55), this represents a limitation of the present study. Future larger-scale studies are warranted to clarify potential sex-specific effects of mild cortisol excess on musculoskeletal health.

On the other hand, the strengths of the study include accurate measurements of muscle strength and physical performance, which are imperative for defining sarcopenia. Furthermore, the presence of three comparison groups, including healthy subjects, is determinant considering recent evidence in the cardiovascular burden of patients with adrenal adenoma (6).

In conclusion, our findings suggest that mild autonomous cortisol secretion may be associated with subtle proximal muscle weakness and increased musculoskeletal burden. However, as this signal was mainly detected by the MRC scale and not consistently across all functional assessments, these results should be interpreted with caution. Given the exploratory nature of the study and the limited sample size, further large-scale studies are needed to confirm these findings and clarify their clinical relevance. Therefore, a comprehensive, multidisciplinary approach in the evaluation of patients with adrenal incidentalomas is meaningful. Clinicians should consider integrating bone health assessments, functional testing, and detailed endocrine evaluations to guide individualized therapeutic strategies. Further large-scale, longitudinal studies are needed to delineate the natural history of MACS, identify high-risk patients, and evaluate the clinical benefit of surgical or pharmacologic interventions to mitigate cortisol-related complications.

## Data Availability

The raw data supporting the conclusions of this article will be made available by the authors, without undue reservation.
