# Supplementary material for: Is muscle strength an overlooked parameter in patients affected by mild autonomous cortisol secretion?
Source: Front Endocrinol (Lausanne). 2026 Apr 30;17:1803910. doi: 10.3389/fendo.2026.1803910 (PMC13171343; doi:10.3389/fendo.2026.1803910)
Supplement: Supplementary file 1 [file Table1.docx]

Supplementary Material

**Supplementary Tables**

**Table S1**. Quality of life and pain assessment results of enrolled patients, divided by groups.

|  | **TOTAL** | **MACS** | **NFAA** | **CONTROL GROUP** | **ANOVA P value** |
| --- | --- | --- | --- | --- | --- |
|  | **(n =62)** | **(n = 21)** | **(n = 21)** | **(n = 20)** |  |
| **EQ-5D movement ability** |  |  |  |  |  |
| No limitations, n (%) | 45 (72.58) | 13 (61.90) | 16 (76.19) | 16 (80.00) | 0.3880^ |
| Some limitations, n (%) | 17 (27.42) | 8 (38.10) | 5 (25.81) | 4 (20.00) |  |
| **EQ-5D personal care** |  |  |  |  |  |
| No limitations, n (%) | 58 (93.33) | 18 (85.71) | 20 (95.24) | 20 (100.00) | 0.5296* |
| Some limitations, n (%) | 3 (4.84) | 2 (9.52) | 1 (4.76) | 0 (0.00) |  |
| Inability to care, n (%) | 1 (1.61) | 1 (4.76) | 0 (0.00) | 0 (0.00) |  |
| **EQ-5D usual activities** |  |  |  |  |  |
| No limitations, n (%) | 46 (74.19) | 13 (61.90) | 15 (71.43) | 18 (90.00) | 0.1528* |
| Some limitations, n (%) | 15 (24.19) | 7 (33.33) | 6 (28.57) | 2 (10.00) |  |
| Inability to perform, n (%) | 1 (1.61) | 1 (4.76) | 0 (0.00) | 0 (0.00) |  |
| **EQ-5D pain** |  |  |  |  |  |
| No pain, n (%) | 19 (30.65) | 5 (23.81) | 8 (38.10) | 6 (30.00) | 0.6377* |
| Some pain, n (%) | 39 (62.90) | 14 (66.67) | 13 (61.90) | 12 (60.00) |  |
| Extreme pain, n (%) | 4 (6.45) | 2 (9.52) | 0 (0.00) | 2 (10.00) |  |
| **EQ-5D depression or anxiety** |  |  |  |  |  |
| Not anxious, n (%) | 33 (52.23) | 12 (57.14) | 8 (38.10) | 13 (65.00) | 0.0944* |
| Moderately anxious, n (%) | 25 (40.32) | 7 (33.33) | 13 (61.90) | 5 (25.00) |  |
| Extremly anxious, n (%) | 4 (6.54) | 1 (9.52) | 0 (0.00) | 2 (10.00) |  |
| **EQ-5D current state of health**, median (Q1-Q3) | 75 (70-80) | 70 (65-80) | 70 (60-85) | 80 (70-85) | 0.1692° |
| **NRS**, median (Q1-Q3) | 4 (0-6) | 4 (4-6) | 4 (0-5) | 2 (0-5) | 0.2019° |

^chi square test, * Fisher's exact test, ° Kruskall-Wallis test,^#^ Mann-Whitney test

**Abbreviations: EQ-5D**: EuroQoL-5D, **NRS:** Numeric Rating Scale

**Table S2**. Medians and interquartile ranges of cortisol levels across demographic and clinical parameters for MACS and NFAA groups

|  | **Median (Q1-Q3)** | **Kruskall-Wallis  p-value** |
| --- | --- | --- |
| **Gender** |  |  |
| **F** | 1.95 (1.50-2.95) | 0.3519 |
| **M** | 1.85 (1.25-2.70) |  |
| **Smoker** |  |  |
| *None* | 1.80 (1.40-2.60) | 0.1160 |
| *Current* | 2.00 (1.10-2.80) |  |
| *Past* | 5.18 (2.95-7.40) |  |
| **Comorbidity** |  |  |
| **T2D** |  |  |
| *No* | 1.95 (1.40-2.80) | 0.8382 |
| *Yes* | 1.80 (1.20-2.00) |  |
| *IFG* | 1.80 (1.30-2.70) |  |
| **Hypertension** |  |  |
| *No* | 1.85 (1.10-2.00) | 0.6280 |
| *Yes* | 1.85 (1.40-2.80) |  |
| **Dyslipidemia** |  |  |
| *No* | 1.90 (1.25-2.85) | 0.7712 |
| *Yes* | 1.85 (1.40-2.70) |  |
| **Bone** |  |  |
| *No* | 1.70 (1.30-2.20) | 0.2098 |
| *Osteoporosis* | 2.00 (1.20-3.50) |  |
| *Osteopenia* | 2.70 (2.30-2.85) |  |
| **Bilateral adrenal adenoma** |  |  |
| *No* | 1.85 (1.45-2.70) | 0.9766 |
| *Yes* | 1.80 (1.10-2.95) |  |
| **MRC SCALE – right biceps brachii** |  |  |
| *Movement possible against moderate resistance* | 2.40 (2.00-2.80) | 0.1477 |
| *Normal force* | 1.75 (1.25-2.75) |  |
| **MRC SCALE - bicipite sx** |  |  |
| *Movement possible against moderate resistance* | 2.40 (1.50-2.80) | 0.6296 |
| *Normal force* | 1.80 (1.35-2.75) |  |
| **MRC SCALE - quadricipite femorale dx** |  |  |
| *Movement possible against gravity but not against resistance* | 1.60 (1.60-1.60) | 0.5278 |
| *Movement possible against moderate resistance* | 2.60 (1.60-2.90) |  |
| *Normal force* | 1.80 (1.30-2.20) |  |
| **MRC SCALE - quadricipite femorale sx** |  |  |
| *Movement possible against gravity but not against resistance* | 1.80 (1.80-1.80) | 0.8460 |
| *Movement possible against moderate resistance* | 1.60 (1.40-2.70) |  |
| *Normal force* | 1.90 (1.20-2.90) |  |
| **SIT TO STAND TEST** |  |  |
| *>16,6’’* | 1.60 (1.00-2.00) | 0.3470 |
| *16,6’’-13,7’’* | 1.80 (1.45-2.80) |  |
| *13,6’’-11,2’’* | 2.50 (1.40-3.50) |  |
| *<11,2’’* | 1.80 (1.10-2.60) |  |
| **EQ-5D movement ability** |  |  |
| *No limitations* | 1.70 (1.40-2.20) | 0.2124 |
| *Some limitations* | 2.20 (1.80-2.90) |  |
| **EQ-5D personal care** |  |  |
| *No limitations* | 1.80 (1.30-2.80) | 0.4400 |
| *Some limitations* | 2.20 (1.50-7.40) |  |
| *Inability to care* | 2.70 (2.70-2.70) |  |
| **EQ-5D usual activities** |  |  |
| *No limitations* | 1.80 (1.35-2.50) | 0.6757 |
| *Some limitations* | 2.00 (1.50-2.80) |  |
| *Inability to perform* | 2.60 (2.60-2.60) |  |
| **EQ-5D pain** |  |  |
| *No pain* | 1.60 (1.40-2.70) | 0.5890 |
| *Some pain* | 1.90 (1.30-2.90) |  |
| *Extreme pain* | 2.40 (2.20-2.60) |  |
| **EQ-5D deprrssion or anxiety** |  |  |
| *Not anxious* | 1.95 (1.45-2.90) | 0.3025 |
| *Moderately anxious* | 1.70 (1.10-2.40) |  |
| *Extremly anxious* | 2.40 (2.20-2.60) |  |

**Abbreviations: MRC scale:** Medical Research Council scale, **EQ-5D**: EuroQoL-5D

**Table S3**. Correlation analysis (Spearman correlation coefficients and related p values) between demographic and clinical variables with post-DST cortisol levels related to MACS and NFAA group.

|  | **Rho** | **p-value** |
| --- | --- | --- |
| **Age,** years | 0.070 | 0.6584 |
| **Calcium,** mg/dl | -0.063 | 0.7073 |
| **25OH Vitamin D,** ng/ml | -0.328 | 0.0671 |
| **BMI,** kg/m^2^ | -0.009 | 0.9563 |
| **LM,** kg | 0.036 | 0.8224 |
| **LM,** % | 0.117 | 0.4612 |
| **MM,** kg | 0.036 | 0.8224 |
| **MM,** % | 0.113 | 0.4749 |
| **ASM,** kg | 0.070 | 0.6618 |
| **ASMI,** kg/m^2^ | 0.041 | 0.7947 |
| **UL-ASM,** kg | 0.060 | 0.7048 |
| **LL-ASM,** kg | 0.073 | 0.6458 |
| **FM,** kg | -0.027 | 0.8635 |
| **FM,** % | -0.110 | 0.4900 |
| **A-FM,** % | 0.048 | 0.7612 |
| **V-FM,** level | 0.034 | 0.8291 |
| Medium level, n (%) | -0.113 | 0.4764 |
| High level, n (%) | 0.043 | 0.7852 |
| Very high level, n (%) | 0.044 | 0.7819 |
| **FM**(%)**/LM**(%) | 0.047 | 0.7700 |
| **Sarcopenic INDEX** (kg/m2) | 0.088 | 0.5803 |
| **Total body water,** kg | -0.037 | 0.8144 |
| **Total body water,** % | 0.041 | 0.7980 |
| **ECW,** kg | 0.037 | 0.8144 |
| **ECW/TBW,** % | -0.049 | 0.7559 |
| **ICW,** KG, median (Q1-Q3) | 0.025 | 0.8732 |
| **DYNANOMETRY value – righ upper arm** | 0.045 | 0.7749 |
| **DYNANOMETRY value – left upper arm** | 0.066 | 0.6787 |
| **SARC-F value** | 0.199 | 0.2068 |
| **EQ-5D current health state** | -0.059 | 0.7108 |
| **NRS** | 0.187 | 0.2366 |

**Abbreviations:**, **BMI**: body mass index, **LM:** lean mass, **MM:** muscle mass, **ASM:** appendicular skeletal mass, **ASMI:** appendicular skeletal mass index, **UL-ASM:** upper limb ASM, **LL-ASM,** lower limb ASM, **FM:** fat mass, **A-FM:** abdominal FM, **V-FM:** visceral FM, **ECW:** extra cellular water, **TBW:** total body water, **EQ-5D**: EuroQoL-5D, **NRS:** Numeric Rating Scale

**Table S4**. Correlation analysis (Spearman correlation coefficients and related p values) between demographic and clinical variables with post-DST cortisol levels related to MACS group.

|  | **rho** | **p-value** |
| --- | --- | --- |
| **Age,** years | -0.022 | 0.9258 |
| **Calcium,** mg/dl | -0.032 | 0.8930 |
| **25OH Vitamin D,** ng/ml | -0.016 | 0.9512 |
| **BMI,** kg/m^2^ | 0.252 | 0.2696 |
| **LM,** kg | 0.341 | 0.1304 |
| **LM,** % | -0.080 | 0.7301 |
| **MM,** kg | 0.341 | 0.1304 |
| **MM,** % | -0.081 | 0.7258 |
| **ASM,** kg | 0.319 | 0.1587 |
| **ASMI,** kg/m^2^ | 0.279 | 0.2213 |
| **UL-ASM,** kg | 0.256 | 0.2636 |
| **LL-ASM,** kg | 0.346 | 0.1244 |
| **FM,** kg | 0.273 | 0.2320 |
| **FM,** % | 0.081 | 0.7258 |
| **A-FM,** % | 0.275 | 0.2269 |
| **V-FM,** level | 0.375 | 0.0935 |
| Medium level, n (%) | 0.081 | 0.7258 |
| High level, n (%) | 0.279 | 0.2200 |
| Very high level, n (%) | 0.328 | 0.1467 |
| **FM**(%)**/LM**(%) | -0.092 | 0.6922 |
| **Sarcopenic INDEX** (kg/m2) | 0.387 | 0.0832 |
| **Total body water,** kg | -0.066 | 0.7749 |
| **Total body water,** % | 0.277 | 0.2247 |
| **ECW,** kg | 0.066 | 0.7749 |
| **ECW/TBW,** % | 0.329 | 0.1453 |
| **ICW,** KG, median (Q1-Q3) | 0.391 | 0.0794 |
| **DYNANOMETRY value – righ upper arm** | 0.098 | 0.6716 |
| **DYNANOMETRY value – left upper arm** | 0.163 | 0.4799 |
| **SARC-F value** | -0.071 | 0.7584 |
| **EQ-5D current health state** | -0.206 | 0.37 |
| **NRS** | 0.102 | 0.6611 |

**Abbreviations:** **BMI**: body mass index, **LM:** lean mass, **MM:** muscle mass, **ASM:** appendicular skeletal mass, **ASMI:** appendicular skeletal mass index, **UL-ASM:** upper limb ASM, **LL-ASM,** lower limb ASM, **FM:** fat mass, **A-FM:** abdominal FM, **V-FM:** visceral FM, **ECW:** extra cellular water, **TBW:** total body water, **EQ-5D**: EuroQoL-5D, **NRS:** Numeric Rating Scale
